# Quantitative Electron Beam‐Single Atom Interactions Enabled by Sub‐20‐pm Precision Targeting

**DOI:** 10.1002/advs.202502551

**Published:** 2025-06-25

**Authors:** Kevin M. Roccapriore, Frances M. Ross, Julian Klein

**Affiliations:** ^1^ Center for Nanophase Materials Sciences Oak Ridge National Laboratory Oak Ridge TN 37830 USA; ^2^ Department of Materials Science and Engineering Massachusetts Institute of Technology Cambridge MA 02139 USA

**Keywords:** 2D materials, atomic manipulation, electron beam positioning, electron microscopy, spectroscopy

## Abstract

The ability to probe and control matter at the picometer scale is essential for advancing quantum and energy technologies. Scanning transmission electron microscopy offers powerful capabilities for materials analysis and modification, but sample damage, drift, and scan distortions hinder single atom analysis and deterministic manipulation. Materials analysis and modification via electron–solid interactions can be transformed by precise delivery of electrons to a specified atomic location, maintaining the beam position despite drift, and minimizing collateral dose. Here a fast, low‐dose, sub‐20‐pm precision electron beam positioning technique is developed, “atomic lock‐on,” (ALO), which offers the ability to position the beam on a specific atomic column *without* previously irradiating that column. This technique is used to lock onto a single selected atomic location to repeatedly measure its weak electron energy loss signal despite sample drift. Moreover, electron beam‐matter interactions in single atomic events are measured with μs time resolution. This enables observation of single‐atom dynamics, such as atomic bistability, revealing partially bonded atomic configurations and recapture phenomena. This opens prospects for using electron microscopy for high‐precision measurements and deterministic control of matter for quantum technologies.

## Graphical abstract

Atomic lock‐on (ALO) is a rapid, low‐dose in situ technique in scanning transmission electron microscopy (STEM) that achieves sub‐20 picometer (pm) beam positioning. A sparse annular scan collects positional information while avoiding irradiation of the target site. This enables beam positioning that compensates for drift and distortions, allowing quantitative beam‐matter interactions to be studied at the atomic scale in STEM. 

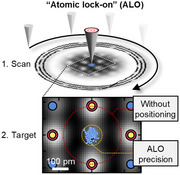

## 1 Introduction

Modern aberration‐corrected scanning transmission electron microscopy (STEM) underpins our understanding of the structure, chemistry and bonding of materials. It enables us to probe atomic arrangements,^[^ ^1^, ^2^, ^3^, ^4^ ^]^ measure elemental identity and electronic configuration through spectroscopy,^[^ ^4^, ^5^, ^6^, ^7^ ^]^ and control matter at the atomic scale by displacing atoms to trigger material reactions.^[^ ^8^, ^9^, ^10^, ^11^, ^12^ ^]^ Controlled nanoscale material modifications are particularly intriguing, since single atomic defects in layered materials can serve as sources of quantum light;^[^ ^13^ ^]^ however, their deterministic generation has not yet been achieved. Despite advances in microscopy technique, several major challenges remain. The interaction of high‐energy electrons with the atoms in the sample often leads to unwanted sample damage.^[^ ^14^ ^]^ Additionally, sample drift and scan distortions during acquisition, even at the Ångström level, complicate signal collection and interpretation.^[^ ^15^, ^16^ ^]^ Overcoming these challenges would revolutionize our capability to analyze materials through imaging and spectroscopy as well as to drive and quantify electron‐solid interactions,^[^ ^14^, ^17^, ^18^ ^]^ ideally at the single‐atom level.

The remarkable measurements of which STEM is capable, such as electron energy loss spectroscopy (EELS) of individual atom columns, are generally achieved by raster scanning a finely focused beam over a sample area and recording data at each point, which is then presented as a map of intensity or energy.^[^ ^19^, ^20^ ^]^ Repeated raster scanning allows dynamic processes to be studied, including amorphization, recrystallization, phase transformations^[^ ^21^, ^22^, ^23^ ^]^ or the movement^[^ ^8^, ^9^, ^24^, ^25^, ^26^, ^27^, ^28^ ^]^ or displacement^[^ ^29^, ^30^, ^31^, ^32^ ^]^ of atoms. However, at the single‐atom level, measuring fast dynamics or highly localized signals requires the ability to systematically target a single atomic column, bond, or defect with the beam, *without* dosing nearby locations. This capability has so far been out of reach, but is fundamental to enabling compositional analysis of specific sites and the manipulation of individual atoms or columns of atoms.

**Figure 1:**
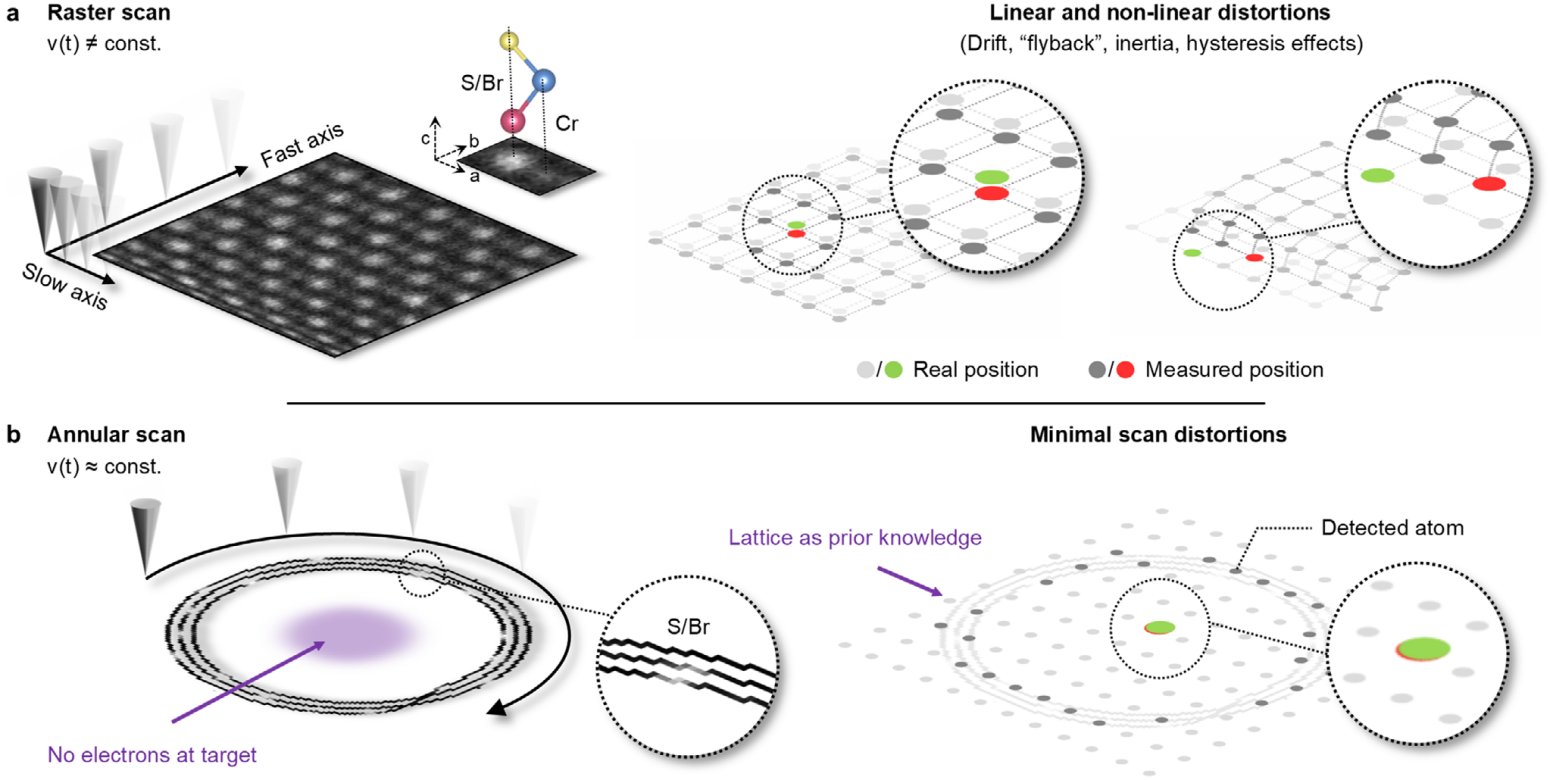
In situ positioning inaccuracy due to linear and nonlinear imaging distortions in STEM. a) Conventional raster scan showing the “fast” and “slow” scan axes and a non‐uniform electron beam velocity (*v*(*t*) ≠ *const*.), illustrated with CrSBr. The real atomic lattice *L*′(*x*, *y*) (light gray) or target atom position (green) deviates from the measured lattice *L*(*x*, *y*) (dark gray) or target atom position (red). b) Annular scan with a near‐constant electron beam velocity (*v*(*t*) ≈ *const*.). The minimization of scan distortions in the annular scan allows the real atomic lattice to be inferred from sparse atom column information using atomic lock‐on because *L*′(*x*, *y*) ∼ *L*(*x*, *y*).

In situ electron beam positioning has been an unsolved challenge mainly because positional information requires exposing the area of interest to a high electron dose. Moreover, conventional raster scanning limits acquisition speed and exacerbates imaging distortions and sample drift (**Figure** **1**a). Sample drift results in a deviation of the measured from the real lattice coordinates, *L*′(*x*, *y*) ≠ *L*(*x*, *y*), while, even more critically, the nonlinear distortions prevalent in raster scans^[^ ^15^, ^16^ ^]^ nontrivially convolve the real lattice coordinates into different measured coordinates *L*′(*x*, *y*) = *f*(*L*(*x*, *y*)), due to the nonuniform beam movement, inertia of the magnetic scan coils at high scan speeds, and abrupt changes in scan direction that take place along the “fast” and “slow” scan axes and during rapid repositioning (“flyback”).

These challenges substantially complicate single atom measurements, particularly when performed manually^[^ ^9^, ^11^ ^]^ or (more recently) assisted by deep convolutional neural networks (DCNNs).^[^ ^33^, ^34^ ^]^ Although atom column positions can be extracted using DCNNs in images, they do not reflect the real position of the site of interest due to the accumulation of scan distortions and drift in the collected image (Figure 1a). Moreover, since all current approaches for positioning require an image to be obtained, the material experiences a high electron dose that can change or damage it, including at the area of interest. Correcting image distortions can be achieved but requires a substantial dose; furthermore, since the correction is applied during post‐processing steps it is not suitable for in situ experiments.^[^ ^35^, ^36^ ^]^ This combination of challenges strongly motivates the development of an in situ electron beam positioning strategy that circumvents the effects of drift, scan‐based distortions and excessive irradiation dose.

We address these challenges using a sparse annular scan pattern (Figure 1b). This has several advantages. First and most critically, the target region receives no dose. Sparse scanning requires significantly fewer total electrons for lattice reconstruction; therefore, it is fast and minimally invasive for the studied material, preventing unintended structural modifications. Second, the radial symmetry and uniform velocity of the electron beam together substantially reduce the scan distortions.^[^ ^37^, ^38^ ^]^ The sparse annular scan parameters are optimized to collect just enough information to reconstruct the lattice structure when combined with a priori structural information of the crystal under study. Using this approach, the measured atomic lattice now matches the real atomic lattice coordinates much more closely, *L*′(*x*, *y*) ∼ *L*(*x*, *y*) (Figure 1b).

**Figure 2:**
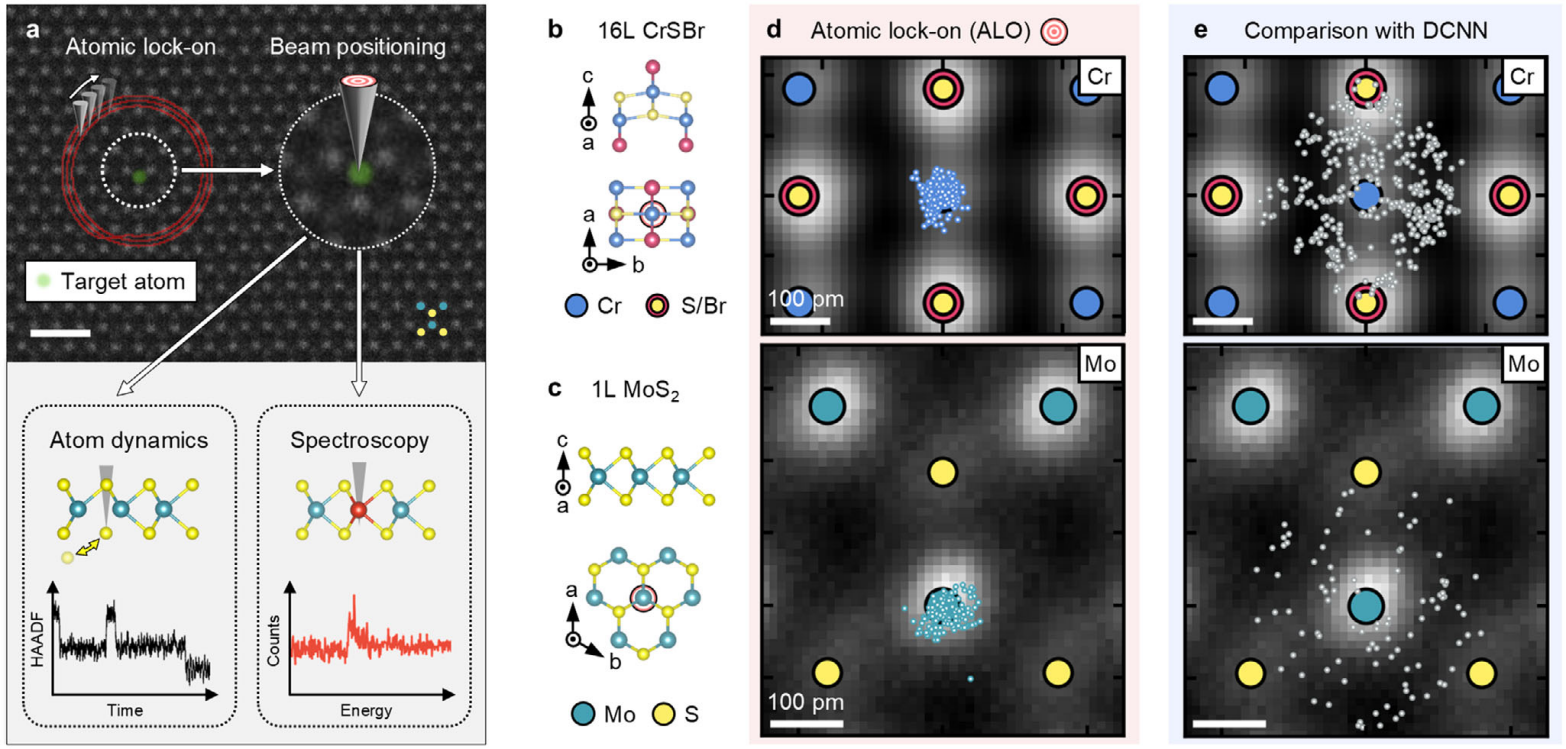
“Atomic lock‐on” for few‐picometer in situ electron‐beam targeting in STEM. a) Illustration summarizing use cases, where precision in atomic targeting enables quantitative single‐atom dynamics and spectroscopy. Scale bar is 1 nm. b) Atomic structure of CrSBr. c) Atomic structure of MoS_2_. d) The accuracy of positioning on a Cr atom column over multiple experiments using atomic lock‐on, showing a precision of 18 ± 10 pm in 16L CrSBr. Targeting the Mo atom in 1L MoS_2_ with atomic lock‐on shows a precision of 29 ± 15 pm in multiple experiments. e) Result of multiple positioning experiments using a deep convolutional neural network (DCNN) with Cr as the target atom column in 16L CrSBr and Mo as target atom in a monolayer (1L) of MoS_2_ both with a precision >100 pm.

We refer to this in situ electron beam targeting technique as “atomic lock‐on” (ALO). We show below that ALO enables positioning of the electron beam on a chosen location of a crystal rapidly, with low dose, and with sub‐20 pm precision. We have developed a workflow that achieves positioning by performing a sparse annular scan followed by a fast lattice reconstruction that makes use of prior knowledge of the target material's crystal structure. Using typical annular scan radii in the range ∼1 nm, we avoid exposing the target area with electrons before the beam is placed accurately on the target, and we require only low dose for the annular scan. After describing the workflow, we show high‐precision STEM experiments that are enabled by ALO (**Figure** **2**a). These include targeted spectroscopic measurements essential for quantitative analytical studies at the single‐atom level, the monitoring and control of material modifications such as the generation of single atomic defects, and the quantification of single‐atom dynamics accessed with high time resolution through targeted beam positioning. The precise beam positioning of ALO reveals single‐atom dynamic phenomena that have not previously been resolved using electron microscopy, such as single atom bistability behavior with the highest temporal resolution so far achieved, 10 μs, limited only by the detector and measurement noise. We suggest that positioning the electron beam in STEM using ALO will offer further exciting possibilities by establishing a pathway towards quantitatively probing and deterministically controlling matter.

We first demonstrate the effectiveness of ALO for beam positioning and compare it to targeting using a DCNN. We perform two experiments, selecting as target sites a Cr atom column in a thick crystal of CrSBr (16 layers (L), ∼13 nm thick), and a Mo atom column in a MoS_2_ monolayer (1L, ∼0.8 nm thick) (Figure 2b,c). We obtain the target position by using either the ALO algorithm or from analyzing a “parent image” using a state‐of‐the‐art DCNN similar to that used in Refs. [33, 34].

After targeting with either method, we then assess the accuracy with which the site was located. This assessment is done by collecting a small (1 nm FOV) spiral high‐angle annular dark field (HAADF)‐STEM image, centered around the beam position, from which the target atom column position is visible (Figure S1, Supporting Information). The distortions in this small area scan are low and allow us to determine the precision from the atom column position. The offset between the desired location and the center of this small image tells us the precision of the targeting process. We repeat the process multiple times to obtain a scatter plot of offset values.

Consistently throughout our experiments using the DCNN, we obtain a spread of beam positions around the Cr target atom in thick CrSBr (Figure 2e), extending up to half a unit cell towards an incorrect atom column. This is expected, since the parent image from which positions are predicted shows effects of the accumulated scan distortions and sample drift. In contrast, we repeatably achieve a robust, sub‐20 picometer precision in targeting the Cr atom column by using ALO (Figure 2d). Even on 1L MoS_2_, the more experimentally challenging sample, we demonstrate repeated precision of sub‐30 pm in targeting a Mo atom (Figure 2d). Multiple attempts using the DCNN again show a broad spread of positions relative to the target site (Figure 1a,b). We attribute the slightly lower precision for ALO in this sample to lower signal‐to‐noise ratio and geometric lattice inhomogeneities in freely suspended monolayers. Table S1 (Supporting Information) summarizes the advantages of in situ beam positioning using ALO.

We therefore conclude that a sparse HAADF‐STEM scan is successful in identifying atomic columns and positioning an electron beam on an individual atomic location. The precision of this positioning is limited by thermal lattice vibrations that introduce dynamic displacements of atoms from their equilibrium positions, blurring the atomic potential seen by the beam. Additionally, the finite probe size, delocalization due to inelastic scattering, and long‐range Coulomb interactions can further reduce spatial precision. The interaction volume and spatial uncertainty are broadened compared to the accuracy of the algorithm, setting a fundamental upper limit for the positioning precision.

The ALO scan typically takes 100 ms at a beam current of 20 pA, resulting in a low dose of *D* = 9.2 · 10^4^ *e* ^−^/Å^2^  at a dose rate of $\dot{D}=9.2\cdot 10^{5}e^{-}$/Å^2^ s (see more details in the Experimental Section). Following the scan, lattice reconstruction requires less than 10 ms, with additional operating system processes accounting for the remainder of a 200 ms “off” time. Throughout this “off” period, the beam is electrostatically blanked to prevent damage to the material. We emphasize that ALO can be performed without prior imaging, as long as the sample is in focus; the lattice can be reconstructed from any scan position.

**Figure 3:**
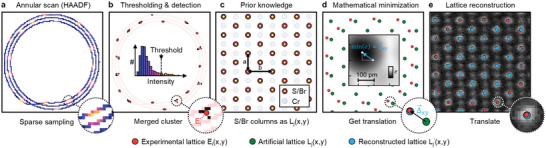
In situ lattice reconstruction via atomic lock‐on, without prior observation of the target site. a) Annular scan and corresponding HAADF detector signal (blue = low counts, yellow = high counts) showing sparsely scanned S/Br atom columns. b) Histogram of HAADF‐STEM scan with threshold filtering used to identify pixels that are in S/Br atom columns. Pixel clusters are merged and the center of mass determines the experimental atom position *E*_*i*_. c) Prior knowledge is used to construct an artificial lattice *L*_*j*_(*x*, *y*) from lattice parameters (a and b) and rotation angle θ. d) Mathematical minimization to obtain the optimal translation vector ${\vec{\Delta }}_{xy}$ that results in the minimum residual *e* (inset) between artificial and experimental lattice. e) The S/Br sub‐lattice is reconstructed by translating the artificial lattice.

**Figure** **3** summarizes the workflow of ALO. Figure 3a shows the sparse HAADF‐STEM annular scan which here consists of three loops and has an outer radius of 1 nm and inner radius of 0.8 nm. This is performed (instead of a full‐area raster scan) immediately before targeting a desired site, and can be repeated as needed during extended experiments over time intervals that depend on the stability of the microscope, the sample, and other factors, as we discuss below. As the beam on its annular path passes over the atom columns they generate a higher detector output signal: for the example of CrSBr, the sub‐lattice of S/Br columns appears the brightest. We then isolate the pixels associated with this sub‐lattice by thresholding the annular scan signal (Figure 3b). We merge clusters of pixels dispersed along the circumferential and radial directions to obtain the experimental lattice positions *E*_*i*_(*x*, *y*) of the sparsely measured S/Br sub‐lattice. We use atomic lattice constants as a priori information (Figure 3c) to generate an artificial lattice *L*_*j*_(*x*, *y*) (Figure 3d). This is followed by a mathematical minimization to find the best overlap of the artificial lattice with the experimental points via residual minimization to obtain the optimal translation vector ${\vec{\Delta }}_{xy}$. In the final step, we add ${\vec{\Delta }}_{xy}$ to the lattice point locations (Figure 3e) to obtain the translated lattice $L_{j}^{\prime }\left(x,y\right)$. Now any position is known by a translation relative to the reconstructed sub‐lattice. This allows us to determine the location of any site within the unit cell without directly observing that site with electrons. In this example we reconstruct the S/Br sub‐lattice, but the approach is independent of crystal symmetry and we have applied it to mono‐ and multi‐atomic compounds combining different atomic numbers, provided the material is periodic. Despite the presence of crystal distortions and defects in monolayers, we find ALO to be robust, attributed to the detection of multiple atoms in the annular scan. This minimizes the effects of defects and distortions, maintaining a sub‐30 pm accuracy even in monolayers (Figure 2d). The precision provided via ALO consequently enables us to repeatedly target individual atoms with the electron beam *without* previously observing them, even in the sensitive monolayer case.

**Figure 4:**
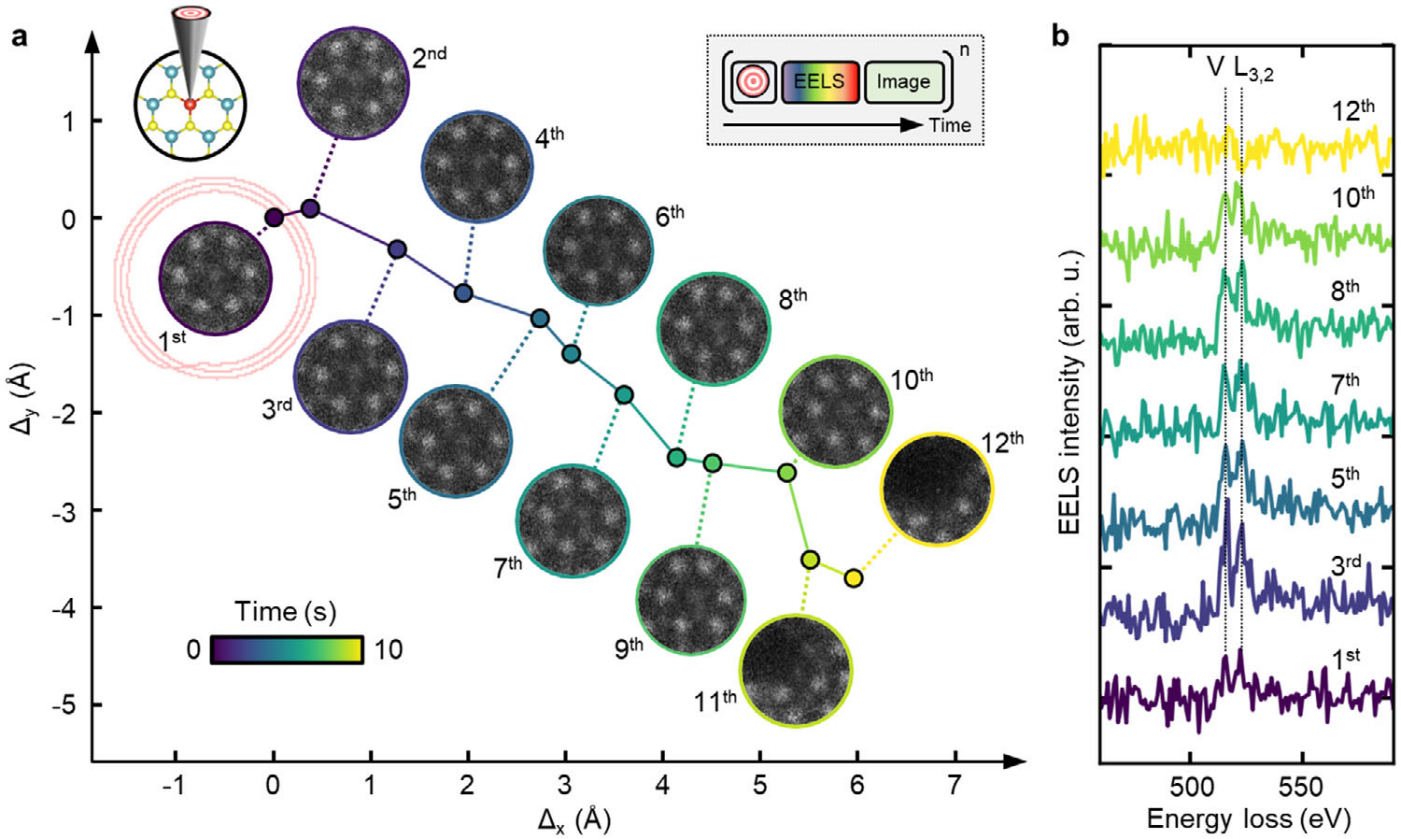
Dynamic targeting and single‐shot localized EELS on a V dopant atom in 1L MoS_2_. a) Time‐dependent tracking performing 12 consecutive atomic lock‐ons on the V dopant atom. The accumulated and compensated drift ${\vec{\Delta }}_{xy}$ is shown in time alongside collected small HAADF‐STEM images. Inset: Automated experimental sequence. b) Corresponding single‐shot EELS spectra collected following atomic lock‐on showing the L_3, 2_ edge of the V dopant atom. The EELS integration time per spectrum was 250 ms at a beam energy of 60keV and a beam current of 20 pA corresponding to 3.12 · 10^7^ *e* ^−^ at a dose of *D* = 6.2 · 10^7^ *e* ^−^/Å^2^  and a dose rate of $\dot{D}=2.5\cdot 10^{8}e^{-}/{Å}^{2}\;s$.

**Figure 5:**
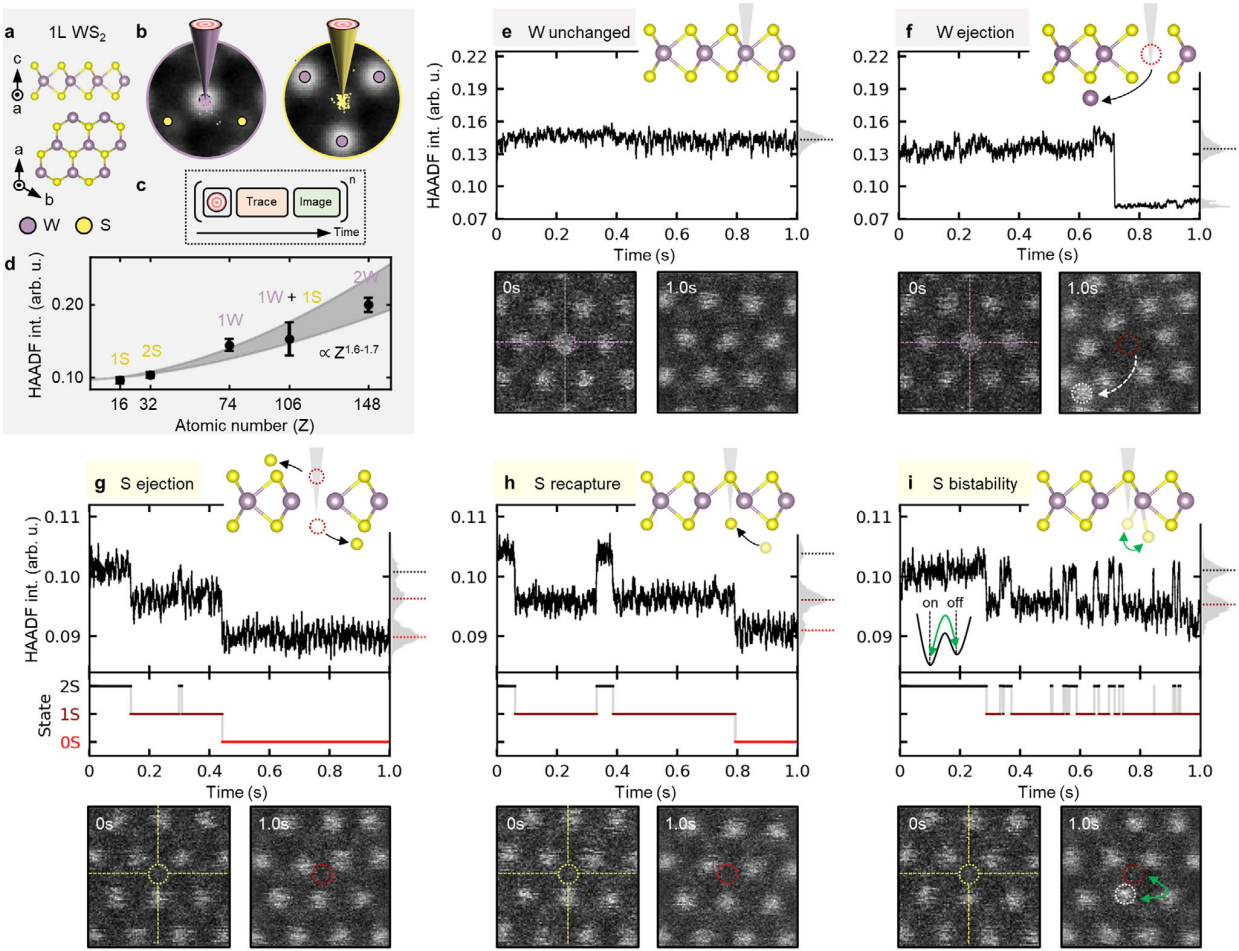
Single‐atom dynamics with sub‐millisecond time resolution in 1L WS_2_. a) 1L WS_2_ crystal structure. b, Atomic lock‐on targeting of the W atom and 2S atom column with 28 ± 18 pm ad 27 ± 17 pm precision, respectively. c) Experimental workflow. d) HAADF intensity as a function of atomic number Z. The dashed grey line is a model ∝ *Z* ^1.6 − 1.7^ to the data. Atomic lock‐on targeting of e–f) W atom column and g–i) 2S atom column for 1 s and corresponding HAADF‐STEM image before and after experiment. The corresponding HAADF intensity histogram depicts different structural states. A moving average of 3 ms was applied to the time traces. A beam energy of 60keV and a beam current of 20 pA were used, corresponding to 1.25 · 10^8^ *e* ^−^ for 1 s at a dose of *D* = 6.2 · 10^7^ *e* ^−^/Å^2^  and a dose rate of $\dot{D}=2.5\cdot 10^{8}e^{-}/{Å}^{2}\;s$.

We systematically optimized the scan parameters for ALO, such as inner and outer radius, number of roundtrips and pixel dwell time, to achieve the highest precision with the least electron dose (Figures S2, S3, and S4, Supporting Information). This involved performing a grid search simulating ALO and then reducing the dwell time to a level where we reconstruct the lattice with < 50 pm precision 100 percent of the time. While ALO works for many choices of inner and outer radii, we found that three roundtrips are necessary for robust positioning. The details of the optimization are discussed in the Supporting Information.

The most challenging of STEM measurements involve probing a specific location in a sample over an extended time, for example to integrate weak spectroscopic signals or measure single‐atom dynamics. We describe below examples of both of these measurements, where the beam is placed on the target with sub‐20pm precision and unnecessary dosing of the target area is avoided. Instead, repeated operation of ALO, which can be carried out in an automated sequence, compensates for sample drift to locate, track and measure one specific atom within a particular unit cell.

By keeping the beam on a single dopant atom for times exceeding 1 second we measure its weak EELS core loss signal. This is shown for a single V dopant atom in 1L MoS_2_ in **Figure** **4**. Quantifying a single dopant atom is a challenging task,^[^ ^19^, ^39^ ^]^ and therefore ideal for this demonstration. Because our target is a single, non‐periodic feature (a V dopant atom), we first must collect a single parent image. (We emphasize that this step is not necessary if the sample is periodic.) We then identify the initial location to be targeted with a pre‐trained DCNN.^[^ ^34^ ^]^ From this point on, ALO is used; we find that the DCNN alone is not sufficiently accurate to provide the correct dopant location. We carry out repeated operations consisting of ALO at the selected dopant atom followed by collection of a single‐shot EEL spectrum from this location with a sampling time of 830 ms. We repeat the lock‐on and spectroscopy 12 times over 10 s. This time interval is chosen based on the drift rate of the microscope: it is important to re‐center the dopant atom before the drift approaches one unit cell, since the annular scan analysis algorithm can not distinguish between unit cells. The value of ${\vec{\Delta }}_{xy}$ obtained in each operation is effectively the (compensated) sample drift in time. Figure 4a shows the values of ${\vec{\Delta }}_{xy}$ obtained during an experiment while Figure 4b shows the EELS spectra obtained after selected ALO operations. Each EELS spectrum shows the *L*_3, 2_ edge with expected energies of 513eV and 521eV. The experiment has therefore successfully tracked the V dopant atom for multiple seconds while compensating for maximum drift rates of >1 Å s^−1^. We have also demonstrated extended operation for even higher drift rates of 2 Å s^−1^ (Figure S5, Supporting Information).

To validate the positioning and observe the environment around the dopant atom, in this experiment we also recorded a small (1 nm FOV) HAADF‐STEM spiral image after each EELS measurement. Acquiring these images is not strictly necessary for the primary measurement, and adds dose to the target area which can influence the local environment. However, the images provide valuable diagnostic information: confirming the stability of the target site during acquisition, monitoring changes in atomic coordination, and identifying transient states. In principle, the dose in such images can be optimized to yield only the essential information, such as the continued presence of the dopant atom. In the images, Figure 4a, the V atom appears as a darker feature compared to Mo. It is successfully tracked for over 8 s. At this time the recorded images change, as the accumulated dose has visibly damaged the material. The spectroscopic and imaging data present opportunities for correlation. The changing intensity ratio and line broadening throughout later iterations (5^*th*^ to 10^*th*^) is suggestive of electronic changes, potentially due to the formation of S vacancy defects visible from lattice distortions. The increasing prevalence of lattice distortions due to repeated measurements have slightly displaced the V dopant atom from the corrected center location of ALO, but it is still close enough to the beam location to provide an EELS signal.

The approach in Figure 4 offers the advantage of significantly reducing the electron dose needed to obtain the same information, compared with grid‐based spectrum imaging techniques (Figure S6, Supporting Information). In techniques where a spectrum is collected from each point and those spectra that came from the area of interest are selected afterwards, there is a greater possibility of inducing structural modifications during acquisition. An intriguing possibility suggested by the approach in Figure 4 is to collect spectroscopy data not only at individual atoms but also at bonds or other specific points within the lattice (Figure S6, Supporting Information). We finally note that this experiment is scalable in the sense that for any material and spectroscopic measurement, the atom can be tracked *without directly observing it* for as long a time as needed via repeated lock‐ons, terminating when sufficient data is collected or the sample becomes damaged.

The ability to position the electron beam precisely on one specific atom then record a detector signal as a function of time offers a pathway to study the interaction between electrons and individual atoms. In **Figure** **5**a we show such an experiment performed on monolayer thick WS_2_ where the HAADF signal is recorded in time. For both the W and the 2S sites, lock‐on obtains consistent sub‐30 pm precision (Figure 5b; Figure S7, Supporting Information). In the automated experiment (Figure 5c), we therefore first perform ALO to position the electron beam on either a W or 2S site. We then monitor the HAADF intensity with a fast sampling frequency of 100 kHz (10μs) for a total time of 1 s. Due to the relationship between HAADF signal and atomic number, we interpret the intensity in terms of the atomic configuration at the target site as a function of time (Figure 5d). As in the spectroscopy experiment, this data acquisition is followed by the collection of a small (1 nm FOV) diagnostic HAADF‐STEM image of the final structure, centered on the target site. We carry out 50 such targeting experiments automatically and sequentially on different W and 2S sites to obtain statistics regarding the range of behavior. A single experiment takes ∼1.5 s resulting in a total experimental acquisition time for each type of target site of ∼75 s. The ability to repeat the measurement quickly makes this approach highly scalable for rapidly collecting statistical data.

We show a selection of recurring experimental behaviors at the W atom site in Figure 5e,f and at the 2S atom site in Figure 5g–i. Additional data is shown in Figure S8 and Figure S9 (Supporting Information). When targeting W, 25% of experiments show no appreciable change in the time‐dependent HAADF intensity (Figure 5e). The final image is also unchanged. In these experiments, positioning the electron beam on a W atom has therefore not led to rearrangement, which can be attributed to the high displacement threshold energy.^[^ ^29^ ^]^ However, 4% of the experiments do show displacement, visible both from the step‐like drop in HAADF intensity (Figure 5e at ∼0.7 s) and from the final image, in which the W atom is displaced and its final location at a neighboring 2S site. Other experiments (Figure S8, Supporting Information) show step‐like changes in the HAADF intensity that may even be an increase over the initial intensity. We attribute these to diffusion of adatoms across the target location. The remaining experiments (making up the majority, 60%) exhibit intensity fluctuations that can be identified as arising from distortions due to defects induced in the 2S sites in proximity to the W atom (Figure S8, Supporting Information).

Targeting the 2S site, Figure 5g–i, is even more interesting because of the lower displacement threshold energy of S^[^ ^29^ ^]^ that results in the formation of a mono‐sulfur vacancy *V*_1*S*_. The experiments frequently (28%) show a signature of S atom ejection and *V*_1*S*_ formation in the form of a step‐like decrease in the HAADF intensity (Figure 5g–i; Figure S9, Supporting Information). In many instances, this is followed by a second drop, directly suggesting the ejection of a second S atom and formation of a di‐sulfur vacancy *V*_2*S*_.

More unexpected is that 66% of the experiments exhibiting an S atom ejection show the *recapture* of S atoms after the initial assumed displacement (Figure 5h at ∼0.35 s; Figure S9, Supporting Information). In the other 34%, we observe random telegraph noise (RTN) (Figure 5k at >0.28 s) between the HAADF intensity levels associated with the 2S and 1S configurations. This suggests fast movement of the S atom between the targeted 2S atom column (“on” state) and a proximal site (“off” state). We interpret the recapture and RTN as indications that the S atom remains partially bonded to the lattice. In this scenario, the energy of an electron is sufficient to drive the transition between the two metastable states (2S↔1S), thereby effectively acting as a bistable atomic system. The shortest duration we can resolve the S atom in the 2S state is <1 ms (<1.2 · 10^5^ *e* ^−^) suggesting even faster time dynamics. Our interpretation is further sgupported by examination of the final HAADF image, which shows increased intensity in one of the three nearest neighbor W sites. Based on the excellent agreement of the intensity at this location with the expected atomic number of 1W + 1S (Figure 5d), we tentatively attribute this intensity to the S atom residing in a potential “off” state.

We have shown two quantitative high‐precision STEM experiments: measuring the weak spectroscopic signal of a single dopant atom with acquisition times of up to a second, even in the presence of sample drift, and characterizing the dynamics of individual atoms with sub‐ms time resolution. These experiments were enabled by precision beam positioning through a rapid algorithm that does not dose the area of interest and can be repeated as needed to locate and track a single atomic site. We showed that ALO can be applied to both thick and thin crystals and we find it particularly promising for challenging beam‐sensitive and atomically thin materials.

The ability to integrate ALO within virtually any STEM workflow, as used in our measurements of beam‐induced displacement, offers exciting possibilities for collection of large numbers of datasets. This enables a statistical quantification of phenomena of interest, which can otherwise be a limitation of traditional STEM observations. We also anticipate that minor algorithm modifications may extend ALO to moderately disordered systems or those with specific structural features (such as twin boundaries), increasing the range of materials that can be examined. For example, ALO could look for multiple characteristic arrangements in a sparse annular scan to accurately determine a phase boundary or interface region and perform positioning on either side. In the experiments described so far, the target location was within the annular scan, but the beam can be positioned far from the scan area, with accuracy depending on intrinsic imperfections in the sample (due to defects, strain fields or sample bending) and the magnetic scan coil hardware and voltage to picometer calibration. Moreover, while ALO enables precise positioning within the xy‐plane, future advances may include mitigating drift in the out‐of‐plane (z) direction.

The high temporal resolution combined with single‐atom sensitivity and real‐time single‐event detection lay the foundation for algorithmic schemes to drive and control atomic modifications. An immediate outcome could be the deterministic generation of single‐atom defects in materials, which is essential for scaling quantum technologies.^[^ ^13^ ^]^ We further anticipate other uses of ALO for in situ STEM studies to observe or control dynamic processes, such as the early stages of nucleation and growth of atomic assemblies. Here, the combination of knowledge of the lattice coordinates and precise positioning of the beam allows single‐atom events to be monitored. The measured intensity provides a natural avenue for end‐point detection. After a displacement event is triggered, as measured by the change in ADF signal, the beam can be repositioned to another atom column. This would be useful if we need to continue acquisition, for example, of EELS signals, or move a series of atoms in minimal time. ALO is applicable generally to crystalline materials, with high Z contrast specimens most readily amenable, but we anticipate its use even for low‐Z materials such as graphene and hexagonal boron nitride, for example if we wish to generate defects. Atomic manipulation by ALO can be synchronized with a range of analytical measurement techniques such as EELS, energy‐dispersive X‐ray spectroscopy or even 4D‐STEM, to perform multimodal studies that obtain chemical and electronic information with minimal disturbance to the local environment. Combining simultaneous data collection with the ability to move between equivalent locations as a function of the evolving atomic configuration could enable acquisition of weak spectroscopic signals, for example for a single S vacancy in MoS_2_ or WS_2_, which is otherwise challenging due to the low signal to noise ratio. Such measurements could benefit experiments where external sample parameters (current, doping density, electric field, light, temperature or even the gas environment) are controlled, for example in catalytic processes. Precise positioning might also be useful to locally inject electrons to trigger single‐defect cathodoluminescence or electron beam‐induced current measurements.

We finally suggest that ALO will open a pathway towards a more systematic understanding of elastic and inelastic scattering mechanisms of individual atoms or columns, their dependence on beam energy and dose rate, and their evolution over different time and energy scales. We anticipate that site‐specific monitoring will help to clarify the complex kinetics of beam‐driven structural phase changes^[^ ^21^, ^22^, ^23^ ^]^ or the effects of beam‐generated secondary electrons. We believe this will contribute to verifying and improving existing classical and quantum mechanical formulations of electron‐beam matter interactions,^[^ ^17^, ^40^ ^]^ refining element‐specific displacement cross sections,^[^ ^29^, ^30^, ^31^, ^32^ ^]^ and studying how the electron beam drives defect formation, migration, and annihilation, with individual atom precision.

## 2 Experimental Section

### Sample Fabrication

Bulk CrSBr flakes were exfoliated using the Scotch tape method onto SiO_2_/Si substrates. Flake thickness was determined by atomic force microscopy and optical phase contrast. Selected flakes were transferred onto S/TEM compatible sample grids using cellulose acetate butyrate (CAB) as polymer handle. After transfer, CAB was dissolved in acetone and the S/TEM grids were rinsed in isopropanol prior to critical point drying. MoS_2_ and WS_2_ were MOCVD and CVD grown and then transferred to S/TEM grids using PMMA and water transfer.

### Scanning Transmission Electron Microscopy

A Nion UltraSTEM operating at either 60 or 200 kV, a nominal probe current of 20 pA, and 32 mrad semiconvergence angle was utilized for the CrSBr, MoS_2_ and WS_2_ atomic lock‐on studies. The HAADF collection angle was 80–200 mrad.

For STEM‐EELS, a Nion Iris spectrometer and direct electron detector (Dectris ELA) were used with a dispersion of 0.90404eV / channel.

Custom scan trajectories were performed using a National Instruments (NI) multifunction I/O field programmable gate array (FPGA, USB‐7856) connected to the external scan input and HAADF output using NI terminal block SCB‐68A. This platform operates on LabVIEW but commands are accessible via Python directly from the microscope user interface, Nion Swift, which allows the user to change annular spiral scan or other parameters.^[^ ^41^ ^]^

An operating field of view (FOV) of 16 nm was used for all experiments. At larger fields of view (e.g., >100 nm), voltage noise on the scan coils can begin to limit positioning precision. On our system (total scan range of ±2.5 V), a 100 × 100 pixel scan over a 2 nm annular ALO scan within a 100 nm FOV corresponds to voltage steps of approximately 1 mV. The scan controller used exhibits a root mean square (RMS) voltage noise of ∼250μV (DC to 1 MHz), which translates to a spatial noise of roughly 5 pm at this scale. Based on this, we estimate that fields of view up to ∼200 nm remain compatible with sub‐20 pm targeting precision using this controller.

### Calculation of Electron Dose and Dose Rate for Atomic Lock‐on

We consider the total trajectory covered during the three loops of the annular scan, and the area of the focused electron beam with a *FWHM* = 0.8 Å. To calculate the dose rate $\dot{D}$ and dose *D*, we start by determining the number of electrons reaching the surface over the total exposure time. For the ALO scan time of 100 ms and for a beam current of 20 pA, the number of electrons, *N*_*e*_, is calculated as $N_{e}=\frac{I\cdot t}{q}=\frac{20\cdot 10^{-12}\;A\cdot 0.1\;s}{1.602\cdot 10^{-19}\;C}\approx 1.248\cdot 10^{7}\;\text{electrons}$. The beam makes three loops with radii 10 Å, 9 Å, and 8 Å. The area covered for one donut is *A*_*donut*_ = π · (*R* ^2^ − *r* ^2^) with $R=\text{radius}+\frac{\text{FWHM}}{2}$ and $r=\text{radius}-\frac{\text{FWHM}}{2}$. The total area covered by the beam is *A*_total_ = *A*_*donut*, 1_ + *A*_*donut*, 2_ + *A*_*donut*, 3_ = 135.72  Å ^2^ . The dose rate is given by $\dot{D}=\frac{1.248\cdot 10^{7}}{135.72\;\;{Å}^{2}\;\cdot 0.1\;s}=9.2\cdot 10^{5}e^{-}$ /Å ^2^s and the corresponding dose for a 100 ms ALO is given by *D* = 9.2 · 10^4^ *e* ^−^/Å ^2^ .

During the time to process the atomic lock‐on scan (∼200 ms), the beam is electrostatically blanked to avoid dosing the material. We note that the atomic lock‐on algorithm itself is fast, <10ms. The atomic lock‐on scan can be performed without obtaining a parent image of the area of interest as it obtains lattice information during the atomic lock‐on scan, assuming a focused condition.

### Calculation of Electron Dose for a Single Spot Exposure

The total electron dose for a time‐dependent single spot exposure is calculated by the total number of electrons in time divided by the effective area of the focused electron beam. We consider a focused electron beam with a diameter equal to the *FWHM* = 0.8 Å resulting in an area of $A=\pi {\left(\frac{\text{FWHM}}{2}\right)}^{2}=0.5\;\;{Å}^{2}$. The total number of electrons deposited to the spot during exposure is $N_{e}=I\cdot t\cdot \frac{1}{e}$. For a beam current of 20 pA and an exposure time of 1 s we determine the total number of electrons of *N*_*e*_ = 1.25 · 10^8^ *e* ^−^. Dividing the total number of electrons *N* by the effective area of the focused beam *A* we obtain an electron dose of 2.5 · 10^8^ *e* ^−^/Å^2^ . For an exposure time of 250 ms we obtain a total number of electrons of *N*_*e*_ = 3.1 · 10^7^ *e* ^−^ at an electron dose of *D* = 6.2 · 10^7^ *e* ^−^/Å^2^  at a dose rate of $\dot{D}=\frac{1.248\cdot 10^{8}}{0.5\;\;{Å}^{2}\;\cdot 1\;s}=2.5\cdot 10^{8}e^{-}/{Å}^{2}\;s$.

### Data Post Processing

No additional post‐processing steps of the HAADF‐STEM images were used to generate the Figures, which are shown as‐acquired and unfiltered.

For the EELS data, a power law background was fitted to the region 420 ‐ 900 eV and was subtracted from each spectrum.

### Neural Network Architecture

Atom detection used in this manuscript for CrSBr, MoS_2_, and WS_2_ and utilized the ensemble learning approach described in Refs. [33, 42]. This consists of DCNNs based on the UNET architecture.^[^ ^43^ ^]^ V dopant detection was based on a previously described learning approach in Ref. [34].

### Copyright Notice

This manuscript has been authored by UT‐Battelle, LLC, under contract DE‐AC05‐00OR22725 with the US Department of Energy (DOE). The US government retains and the publisher, by accepting the article for publication, acknowledges that the US government retains a nonexclusive, paid‐up, irrevocable, worldwide license to publish or reproduce the published form of this manuscript, or allow others to do so, for US government purposes. DOE will provide public access to these results of federally sponsored research in accordance with the DOE Public Access Plan (https://www.energy.gov/doe‐public‐access‐plan).

## Conflict of Interest

The authors declare no conflict of interest.

## Author Contributions

J.K. conceptualized the project, developed and tested atomic lock‐on, K.M.R. and J.K. implemented atomic lock‐on and conducted STEM imaging, J.K. prepared the samples, J.K. and K.M.R. analyzed the experimental data, K.M.R., F.M.R. and J.K. designed the experiments and discussed the results. J.K. wrote the manuscript with input from all co‐authors.

## Supporting information

Supporting Information

Supplemental Video 1

Supplemental Video 2

Supplemental Video 3

Supplemental Video 4

Supplemental Video 5

Supplemental Video 6

Supplemental Video 7

Supplemental Video 8

Supplemental Video 9

Supplemental Video 10

Supplemental Video 11

Supplemental Video 12

Supplemental Video 13

## Data Availability

The data that support the findings of this study are available from the corresponding author upon reasonable request.

## References

[1] P. E. Batson , N. Dellby , O. L. Krivanek , Nature 2002, 418, 617. 12167855 10.1038/nature00972

[2] R. Erni , M. D. Rossell , C. Kisielowski , U. Dahmen , Phys. Rev. Lett. 2009, 102, 9. 10.1103/PhysRevLett.102.096101 19392535

[3] W. Zhou , X. Zou , S. Najmaei , Z. Liu , Y. Shi , J. Kong , J. Lou , P. M. Ajayan , B. I. Yakobson , J.‐C. Idrobo , Nano Lett. 2013, 13, 2615. 23659662 10.1021/nl4007479

[4] O. L. Krivanek , T. C. Lovejoy , N. Dellby , T. Aoki , R. W. Carpenter , P. Rez , E. Soignard , J. Zhu , P. E. Batson , M. J. Lagos , R. F. Egerton , P. A. Crozier , Nature 2014, 514, 209. 25297434 10.1038/nature13870

[5] O. L. Krivanek , M. F. Chisholm , V. Nicolosi , T. J. Pennycook , G. J. Corbin , N. Dellby , M. F. Murfitt , C. S. Own , Z. S. Szilagyi , M. P. Oxley , S. T. Pantelides , S. J. Pennycook , Nature 2010, 464, 571. 20336141 10.1038/nature08879

[6] K. Suenaga , M. Koshino , Nature 2010, 468, 1088. 21160475 10.1038/nature09664

[7] W. Zhou , M. D. Kapetanakis , M. P. Prange , S. T. Pantelides , S. J. Pennycook , J.‐C. Idrobo , Phys. Rev. Lett. 2012, 109, 20. 10.1103/PhysRevLett.109.206803 23215517

[8] P. E. Batson , Microsc. Microanal. 2007, 14, 89. 18096100 10.1017/S1431927608080197

[9] T. Susi , J. Kotakoski , D. Kepaptsoglou , C. Mangler , T. C. Lovejoy , O. L. Krivanek , R. Zan , U. Bangert , P. Ayala , J. C. Meyer , Q. Ramasse , Phys. Rev. Lett. 2014, 113, 11. 10.1103/PhysRevLett.113.115501 25259987

[10] T. Susi , J. C. Meyer , J. Kotakoski , Ultramicroscopy 2017, 180, 163. 28284704 10.1016/j.ultramic.2017.03.005

[11] M. Tripathi , A. Mittelberger , N. A. Pike , C. Mangler , J. C. Meyer , M. J. Verstraete , J. Kotakoski , T. Susi , Nano Lett. 2018, 18, 5319. 29945442 10.1021/acs.nanolett.8b02406 PMC6089495

[12] O. Dyck , M. Ziatdinov , D. B. Lingerfelt , R. R. Unocic , B. M. Hudak , A. R. Lupini , S. Jesse , S. V. Kalinin , Nat. Rev. Mater. 2019, 4, 497.

[13] A. R.‐P. Montblanch , M. Barbone , I. Aharonovich , M. Atatüre , A. C. Ferrari , Nat. Nanotechnol. 2023, 18, 555. 37322143 10.1038/s41565-023-01354-x

[14] R. Egerton , P. Li , M. Malac , Micron 2004, 35, 399. 15120123 10.1016/j.micron.2004.02.003

[15] H. von Harrach , Ultramicroscopy 1995, 58, 1.

[16] L. Jones , P. D. Nellist , Microsc. Microanal. 2013, 19, 1050. 23673234 10.1017/S1431927613001402

[17] F. J. García de Abajo , Rev. Mod. Phys. 2010, 82, 209.

[18] T. Susi , J. C. Meyer , J. Kotakoski , Nat. Rev. Phys. 2019, 1, 397.

[19] Q. M. Ramasse , C. R. Seabourne , D.‐M. Kepaptsoglou , R. Zan , U. Bangert , A. J. Scott , Nano Lett. 2013, 13, 4989. 23259533 10.1021/nl304187e

[20] S. Wang , H. Li , H. Sawada , C. S. Allen , A. I. Kirkland , J. C. Grossman , J. H. Warner , Nanoscale 2017, 9, 6417. 28463370 10.1039/c7nr01127j

[21] P. Y. Huang , S. Kurasch , J. S. Alden , A. Shekhawat , A. A. Alemi , P. L. McEuen , J. P. Sethna , U. Kaiser , D. A. Muller , Science 2013, 342, 224. 24115436 10.1126/science.1242248

[22] Y.‐C. Lin , D. O. Dumcenco , Y.‐S. Huang , K. Suenaga , Nat. Nanotechnol. 2014, 9, 391. 24747841 10.1038/nnano.2014.64

[23] J. Klein , T. Pham , J. D. Thomsen , J. B. Curtis , T. Denneulin , M. Lorke , M. Florian , A. Steinhoff , R. A. Wiscons , J. Luxa , Z. Sofer , F. Jahnke , P. Narang , F. M. Ross , Nat. Commun. 2022, 13, 1. 36109520 10.1038/s41467-022-32737-8 PMC9478124

[24] M. Isaacson , D. Kopf , M. Utlaut , N. W. Parker , A. V. Crewe , Proc. Natl. Acad. Sci. USA 1977, 74, 1802. 16592396 10.1073/pnas.74.5.1802 PMC431007

[25] C. O. Girit , J. C. Meyer , R. Erni , M. D. Rossell , C. Kisielowski , L. Yang , C.‐H. Park , M. F. Crommie , M. L. Cohen , S. G. Louie , A. Zettl , Science 2009, 323, 1705. 19325110 10.1126/science.1166999

[26] J. Lee , W. Zhou , S. J. Pennycook , J.‐C. Idrobo , S. T. Pantelides , Nat. Commun. 2013, 4, 1. 10.1038/ncomms2671 23552065

[27] J. Kotakoski , C. Mangler , J. C. Meyer , Nat. Commun. 2014, 5, 1. 10.1038/ncomms4991 PMC4050261 24874455

[28] H. Li , S. Wang , H. Sawada , G. G. D. Han , T. Samuels , C. S. Allen , A. I. Kirkland , J. C. Grossman , J. H. Warner , ACS Nano 2017, 11, 3392. 28256826 10.1021/acsnano.7b00796

[29] H.‐P. Komsa , J. Kotakoski , S. Kurasch , O. Lehtinen , U. Kaiser , A. V. Krasheninnikov , Phys. Rev. Lett. 2012, 109, 3. 10.1103/PhysRevLett.109.035503 22861869

[30] J. C. Meyer , F. Eder , S. Kurasch , V. Skakalova , J. Kotakoski , H. J. Park , S. Roth , A. Chuvilin , S. Eyhusen , G. Benner , A. V. Krasheninnikov , U. Kaiser , Phys. Rev. Lett. 2012, 108, 19. 10.1103/PhysRevLett.108.196102 23003063

[31] S. Kretschmer , T. Lehnert , U. Kaiser , A. V. Krasheninnikov , Nano Lett. 2020, 20, 2865. 32196349 10.1021/acs.nanolett.0c00670

[32] C. Speckmann , J. Lang , J. Madsen , M. R. A. Monazam , G. Zagler , G. T. Leuthner , N. McEvoy , C. Mangler , T. Susi , J. Kotakoski , Phys. Rev. B 2023, 107, 9.

[33] K. M. Roccapriore , M. G. Boebinger , O. Dyck , A. Ghosh , R. R. Unocic , S. V. Kalinin , M. Ziatdinov , ACS Nano 2022, 16, 17116. 36206357 10.1021/acsnano.2c07451

[34] K. M. Roccapriore , R. Torsi , J. Robinson , S. Kalinin , M. Ziatdinov , Sci. Adv. 2024, 10, 29. 10.1126/sciadv.adn5899 PMC466940 39018401

[35] X. Sang , J. M. LeBeau , Ultramicroscopy 2014, 138, 28. 24444498 10.1016/j.ultramic.2013.12.004

[36] C. Ophus , J. Ciston , C. T. Nelson , Ultramicroscopy 2016, 162, 1. 26716724 10.1016/j.ultramic.2015.12.002

[37] X. Sang , A. R. Lupini , R. R. Unocic , M. Chi , A. Y. Borisevich , S. V. Kalinin , E. Endeve , R. K. Archibald , S. Jesse , Adv. Struct. Chem. Imaging 2016, 2, 1.

[38] X. Sang , A. R. Lupini , J. Ding , S. V. Kalinin , S. Jesse , R. R. Unocic , Sci. Rep. 2017, 7, 1.28127051

[39] A. W. Robertson , Y.‐C. Lin , S. Wang , H. Sawada , C. S. Allen , Q. Chen , S. Lee , G.‐D. Lee , J. Lee , S. Han , E. Yoon , A. I. Kirkland , H. Kim , K. Suenaga , J. H. Warner , ACS Nano 2016, 10, 10227. 27934090 10.1021/acsnano.6b05674

[40] A. Yoshimura , M. Lamparski , J. Giedt , D. Lingerfelt , J. Jakowski , P. Ganesh , T. Yu , B. G. Sumpter , V. Meunier , Nanoscale 2023, 15, 1053. 35703316 10.1039/d2nr01018f

[41] X. Sang , A. R. Lupini , R. R. Unocic , M. Chi , A. Y. Borisevich , S. V. Kalinin , E. Endeve , R. K. Archibald , S. Jesse , Adv. Struct. Chem. Imaging 2016, 2, 1.

[42] A. Ghosh , B. G. Sumpter , O. Dyck , S. V. Kalinin , M. Ziatdinov , npj Comput. Mater. 2021, 7, 1.

[43] O. Ronneberger , P. Fischer , T. Brox , U‐Net: Convolutional Networks for Biomedical Image Segmentation , Springer International Publishing, ISBN 9783319245744, 2015, pp. 234–241.

