# Supplementary material for: Quantitative Electron Beam‐Single Atom Interactions Enabled by Sub‐20‐pm Precision Targeting
Source: Adv Sci (Weinh). 2025 Jun 25;12(34):e02551. doi: 10.1002/advs.202502551 (PMC12442701; doi:10.1002/advs.202502551)
Supplement: Supplementary file 1 — Supporting Information [file ADVS-12-e02551-s009.pdf]

ADVANCED SCIENCE
Open Access

Supporting Information

for *Adv. Sci.*, DOI 10.1002/advs.202502551

Quantitative Electron Beam-Single Atom Interactions Enabled by Sub-20-pm Precision Targeting

Kevin M. Roccapriore, Frances M. Ross and Julian Klein**

Supplemental Information:

Quantitative electron beam-single atom interactions enabled by sub-20-pm precision targeting

Kevin M. Roccapriore,[1,*] Frances M. Ross,[2] and Julian Klein[2,†]

[1] *Center for Nanophase Materials Sciences, Oak Ridge National Laboratory, Oak Ridge, TN, 37830, USA*

[2] *Department of Materials Science and Engineering,*

Massachusetts Institute of Technology, Cambridge, MA 02139, USA

Contents

1. Atomic Lock-On and Deep Convolutional Neural Network (DCNN) Targeting Precision 2

2. Grid search optimization for 16L CrSBr and 1L MoS_2 4

3. Additional Details of Atomic Lock-On 6

3.1. Thresholding Condition 6

3.2. A Priori Information for Lattice Reconstruction 8

3.3. Relative Targeting 8

4. Automated and dynamic drift compensation. 9

5. Dose-Effectiveness of Atomic Lock-On. 9

6. Atomic Lock-On Precision for 1L WS_2 11

7. Additional Single-Atom Time Dynamics. 12

References 14

1. Atomic Lock-On and Deep Convolutional Neural Network (DCNN) Targeting Precision

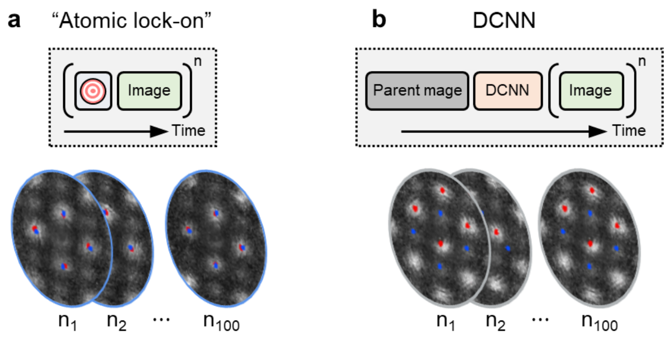

Figure S1. **Experimental comparison between atomic lock-on and a DCNN. a**, HAADF-STEM spiral images (1nm FOV) after ALO targeting of the Cr atom column. Red dots are the four nearest neighbor fitted S/Br atom columns which are used to obtain the offset of the Cr atom position from the center of the spiral image. The blue dots are the ideal positions of the S+Br atom columns for perfect targeting. **b**, HAADF-STEM spiral images (1nm FOV) after DCNN targeting of the Cr atom column.

Figure S1a ,b depict how we determine and compare the targeting precision between ALO and using a deep convolutional neural network (DCNN). For each approach, we obtain the position of the target site (e.g., an atom species) and collect a spiral HAADF-STEM image (1nm FOV) centered on the assumed target position. Experimentally, to test the DCNN, we integrate this procedure into an *in situ* workflow that first takes an overview 'parent image' at low magnification (FOV = 16 nm) from which atomic column positions are predicted using a DCNN trained on MoS_2, WS_2 or CrSBr. In the example of CrSBr, in a subsequent step, a subset of detected Cr atom columns are used as targets, and 25 spiral images are collected. This process is repeated four times to obtain a total of 100 spiral images. To verify the precision, after we perform ALO, we collect a small spiral image with an FOV of 1 nm and high pixel density (10 pm/pixel), from which we determine the offset of the Cr atom column from the center of the image by fitting the four nearest neighbor S/Br atom columns. We repeatedly perform ALOs at different locations after each lattice reconstruction. We fit the next nearest-neighbor S/Br atom columns from which we determine the offset of the atom column from the center (**Fig. S1a, b**). The reported targeting precision refers to the statistical accuracy with which the electron probe can be positioned at a desired location, considering system stability and drift correction. This is distinct from the interaction spread, which is determined by the physical size of the focused electron probe (about 80 pm FWHM). While targeting precision defines how reproducibly we can position the beam, the interaction spread describes the spatial region over which the electron beam interacts with the sample. Both parameters are critical as precise targeting ensures control over beam

placement, while the probe size sets the fundamental spatial resolution limit for interactions such as atomic excitation or displacement.

**Feature**	**Current work: Atomic lock-on (ALO) technique (in situ)**	**Previous work: DCNN-based approach (in situ)**	**General image precision (ex situ)**
1. Ability to measure the position of an atom	-	-	few-pm [1]
2. Ability to place the beam on an atom	**Accurate $<$20pm**	**Inaccurate $>$100pm**	-
3. Electron dose to atom of interest	Atom **remains undosed** until measurement	Atom **has been dosed** prior to measurement	-
4. Image requirement	No pre-acquired image necessary (works blind)	Requires pre-acquired (raster-)scanned image	-
5. Distortion and drift compensation	Actively compensates for distortion and drift through continuous acceleration in annular scanning	Ignores distortion and drift (based on static, pre-acquired image)	-
6. Atom position source	Positions derived directly from sparse annular (spiral) beam motion, ensuring real-time accuracy	Positions determined via DCNN analysis of a (raster-)scanned image	-
7. Dosing in region of interest (ROI)	Minimal, targeted dosing only to essential subregions, minimizing unnecessary exposure	All atoms within ROI receive electron dose	-
8. Capability for Time-Resolved Studies	Enables time-resolved studies by preserving the atom of interest, avoiding cumulative exposure	-	Not suitable for tracking single atoms over time due to continuous dosing

Table S1. Comparison between general *ex situ* atom position precision and *in situ* ALO and DCNN based beam positioning.

Table S1 summarizes the advantages of in situ electron beam positioning of ALO compared to DCNN based positioning. This contrasts with the precision of obtaining the atom position ex situ from a collected image in post-processing. Unlike DCNN, which requires a pre-acquired raster-scanned image and is unable to compensate for distortion or drift, ALO actively compensates through continuous beam acceleration,

sparse and fast annular scanning. Importantly, no pre-acquired image is needed. ALO derives atom positions directly from sparse annular scans, ensuring real-time accuracy while preserving the atom of interest by avoiding unnecessary electron dosing prior to measurement. By targeting only essential subregions for dosing, ALO minimizes cumulative exposure, making it suitable for time-dependent studies and enabling minimally invasive observations of single atoms over time. In comparison, ex situ determination of an atom position from a static image gives a precision of a few picometer [1].

2. Grid search optimization for 16L CrSBr and 1L MoS_2

We perform a grid search optimization to obtain the optimal annular scan shape. Our motivation is to determine the annular scan shape with the highest precision and the absence of failures during repeated operations. Our reasoning for this optimization is that the ideal annulus depends on the crystal symmetry and lattice parameters. For the grid search, we consider two different materials, multilayer (16) CrSBr (**Fig. S2** and monolayer (1L) MoS_2 (**Fig. S3**). We reduce the annulus to three main experimental parameters. In other words, the number of loops N, outer radius r_{out} and inner radius r_{in} of the loops (**Fig. S2a**). To obtain the optimal annulus shape, a grid search was performed. For this, we vary N, r_{out} and r_{in}. For each configuration, we generate 1000 random positions, where the annulus is centered on each of these positions (**Fig. S2b**) and perform ALO to obtain the translated lattice positions L_j (**Fig. S2c**). After each ALO, we compare L_j to the ground truth, which is provided by our HAADF-STEM image (**Fig. S2d**). We use a DCNN to predict the atom column positions that are refined in subsequent steps by performing 2D Gaussian fits. From a comparison with the ground truth, we obtain the offset vector, which is our measure of precision. The offset obtained with respect to the ground-truth lattice are shown in **Fig. S2e**. We obtain a mean precision of $\sim 5\,\mathrm{pm}$. This value is comparable to the Gaussian fitting error of the atom columns ($\sim 2\,\mathrm{pm}$), reflecting the high effectiveness and precision of ALO.

We now perform a grid search for different numbers of loops $N = [1, 2, 3]$ while varying r_{out} and r_{in} (**Fig. S2f, g**) to simulate the mean precision for 304 annular scan shapes for each N. We obtain the best results for $N = 3$ for different combinations of r_{out} and r_{in}. This suggests a fully robust approach and provides flexibility in choosing experimental values depending on the application. Moreover, we find that $N = 2$ is still robust, albeit with slightly lower precision, whereas a single loop $N = 1$ not only provides reduced precision but also exhibits failures, as highlighted in **Fig. S2i**. Therefore, for our experiments, we use $N = 3$ as the ideal trade-off between the highest precision and lowest dose that performs best in challenging experimental environments. For CrSBr, we determine the ideal optimal annular parameters as $r_{out} = 1\,\mathrm{nm}$ and $r_{in} = 0.8\,\mathrm{nm}$.

Similar to the optimal annulus used for CrSBr, we also optimize the scan parameters of the annular

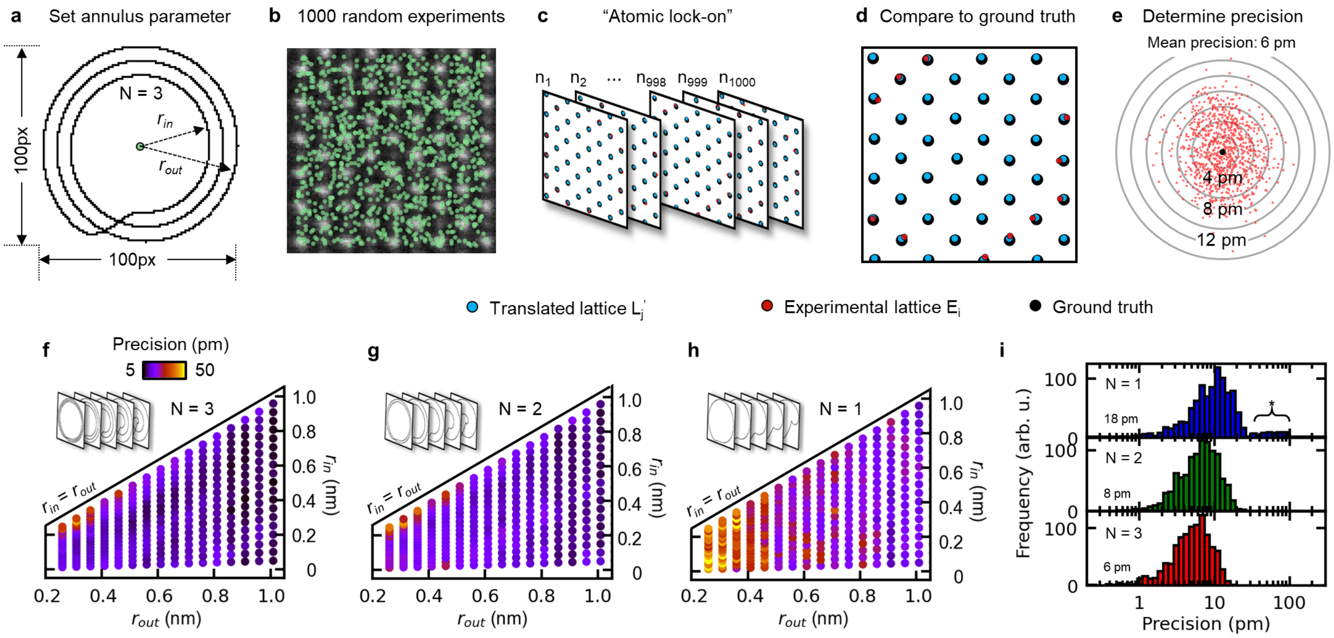

Figure S2. Determining optimal annular scan parameters using grid search simulation for 16L CrSBr. a, Annular scan pattern, defined by three parameters, the outer radius r_{out}, inner radius r_{in} and number of loops N here $N = 3$. Pixel density is $(100px)^2$ and is kept constant. **b**, 1000 random coordinates to statistically evaluate performance of the annular scan at different coordinates. **c**, 1000 ALO simulations at 1000 random coordinates. **d**, Comparison of the reconstructed lattice L_j' with the ground truth lattice positions obtained form a DCNN and 2D Gaussian refinement for obtaining the offset coordinate. **e**, Distribution of offset coordinates with root mean square of $\sim$ 5pm using $r_{out} = 1\,$nm, $r_{in} = 0.8\,$nm and $N = 3$. **f-h**, Grid search simulating a total of 912 annular scan shapes for $N = 3$, $N = 2$, and $N = 1$, respectively. Each point originates from 1000 simulations. **i**, Histogram showing the simulated precision for $r_{in} = 0.8\,$nm and outer radius $r_{out} = 1\,$nm. For each histogram, 1000 random positions were simulated. A single loop ($N = 1$) exhibits failures (highlighted with the *) of ALO absent for $N > 1$.

scan for 1L MoS_2 performing a grid search optimization (**Fig. S3**). We use an experimentally collected HAADF-STEM image for our simulation and define random positions for every set of scan parameters to obtain statistics on the precision. For each of the random positions we perform ALO and determine the lattice parameters based on the obtained translation. We compare this lattice to the ground truth that we obtain from applying a DCNN to the image in combination with a 2D Gaussian atom position refinement step. From the statistics for each set of parameters we obtain a histogram and a precision value. We vary the inner r_{in} and outer r_{out} annulus radius and the number of loops as shown in **Fig. S3a-c**. We observe sets of parameters that are favorable to obtain a consistently high precision. We further compare three histograms

with 1000 random positions for ALO with $r_{\text{in}} = 0.7$ nm and $r_{\text{out}} = 1$ nm and different numbers of loops (**Fig. S3d-f**). We find that a single loop exhibits failures while for $N > 1$ we obtain a mean precision of ~ 13 pm and robust operation.

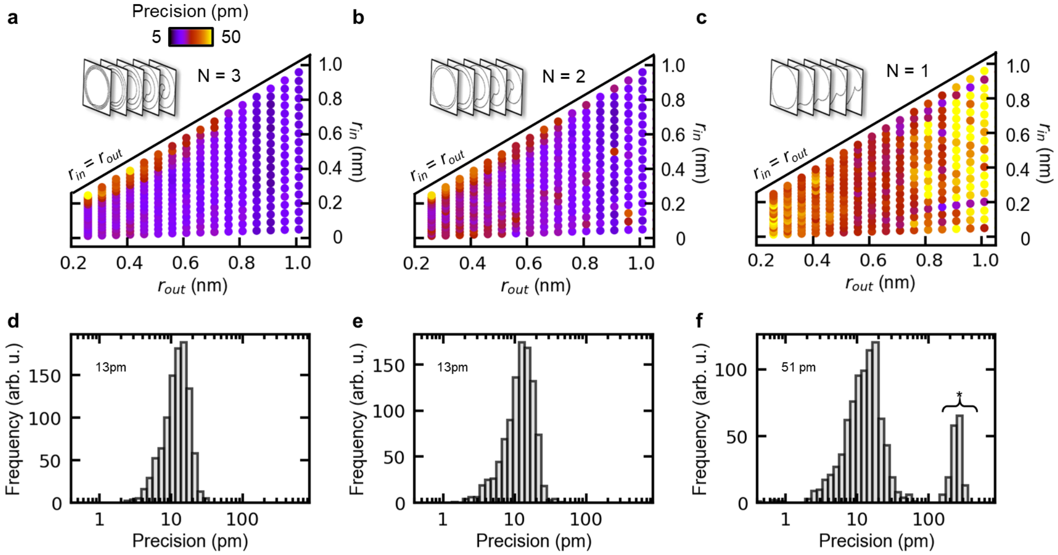

Figure S3. **Grid search optimization for 1L MoS_2 a-c**, Grid search simulating a total of 912 annular scan shapes for $N = 3$, $N = 2$, and $N = 1$, respectively. Each point represents the precision obtained from 1000 random experiments. The precision is shown as a function of inner r_{in} and outer radius r_{out} for different number of loops N of the annulus. **d-f**, Histogram showing the simulated precision for $r_{in} = 0.7\,\text{nm}$ and outer radius $r_{out} = 1\,\text{nm}$. For each histogram, 1000 random positions were simulated. A single loop ($N = 1$) exhibits failures (highlighted with the *) of ALO absent for $N > 1$.

3. Additional Details of Atomic Lock-On

3.1. Thresholding Condition

To isolate the pixels associated with the sub-lattice of interest, we threshold the HAADF-STEM annular scan. There are two options to threshold: either using a defined percentage of all pixels based on the expected coverage of pixels of a target atom column in the sparse scan (geometrical argument), or by using the mean intensity of the target atom column of interest. This can be obtained either by manual input or by using a DCNN to detect atom columns in an image.

Here, we applied a DCNN and obtained the atom column intensity distribution from all the atom columns within a HAADF-STEM image with a field of view (FOV) of 16 nm. With respect to this distribution, and

in the specific example of CrSBr where we detect the S/Br sub-lattice, we set typical threshold values in the range of 90-120% with respect to the overall distribution of S/Br columns, depending on the electron dose used for the scan.

The threshold value (or threshold window) depends on the material and noise conditions of the data, such as the pixel size and pixel dwell time. We quantify the precision of ALO based on the set threshold level (**Fig. S4**). For this, we collect experimental HAADF-STEM images at varying electron doses σ_e and perform 1000 ALOs at random positions to obtain the corresponding mean precision while varying the threshold value (**Fig. S4a**). As expected, we find that the onset for obtaining the best precision moves to lower threshold values for increased doses, owing to the narrower atom column intensity distribution (**Fig. S4b**).

We find that values around 120% are robust for all doses. Moreover, the precision remains high even for very low electron dosages and can be further decreased, which reduces the number of electrons deposited on the sample and the time required to perform the annular scan. This reduces collateral damage and allows for even higher sampling rates for repeatedly using ALO.

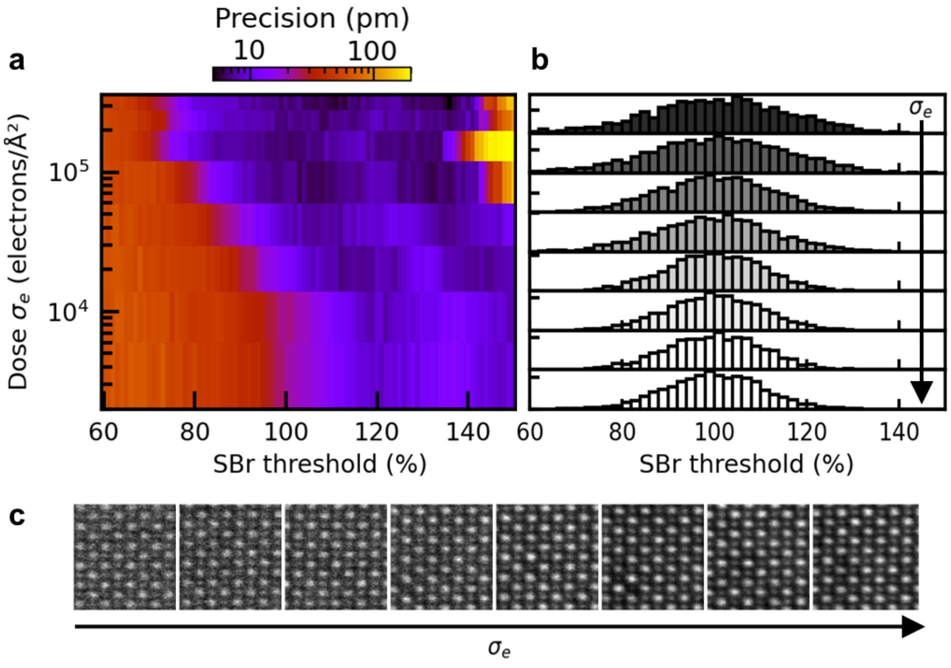

Figure S4. **Atomic lock-on precision for different electron doses. a**, Simulated mean precision as a function of electron dose σ_e and the percentage of the atom column threshold distribution from a DCNN exemplified with the S/Br atom columns in 16L CrSBr. **b**, Intensity distribution of S/Br atom columns with respect to mean intensity obtained from a DCNN. **c**, HAADF-STEM images of CrSBr collected for increasing electron dosages.

3.2. A Priori Information for Lattice Reconstruction

To perform the lattice reconstruction, an artificial sub-lattice is generated, which is optimized onto the experimental lattice obtained from the thresholding. To generate the sub-lattice, lattice parameters such as lattice vectors, orientation, and rotation are required as input. This information can either be obtained from user input, from detecting the reflections in the fast Fourier transform (FFT) of a HAADF-STEM image, or by using a DCNN that predicts the lattice in a collected HAADF-STEM image. In this study, we successfully tested all three options.

In the next step, we perform a mathematical minimization to determine the best overlap of the artificial lattice positions with the experimental lattice positions. For this, we apply a translation vector $\vec{\Delta}_{xy}$ to all lattice positions. To calculate the residual e for a given translation, we translate the lattice positions as follows

$$L'_j = L_j + \vec{\Delta}_{xy} \quad \text{for all } j \tag{1}$$

where L'_j represents the translated lattice positions. Next, we calculate the distance for each experimental position E_i to the closest translated lattice point L'_j using the Euclidean distance

$$D_i = \min_j \left(\sqrt{(E_{ix} - L'_{jx})^2 + (E_{iy} - L'_{jy})^2} \right) \tag{2}$$

where E_{ix} and E_{iy} are the x and y coordinates of the experimental position E_i, and L'_{jx} and L'_{jy} are the x and y coordinates of the translated lattice point L'_j. The residual is obtained as

$$e = \sum_i D_i^2 \tag{3}$$

The optimal translation vector $\vec{\Delta}_{xy}$ is defined by

$$\min(e) = \vec{\Delta}_{xy} \tag{4}$$

3.3. Relative Targeting

In the final step, we add the translation vector to the artificial lattice positions to obtain the actual lattice positions L'_j. In the example of CrSBr, ALO provides the lattice information of the S/Br sub-lattice. To target a specific column in CrSBr, we first select a target site, for example, the Cr atom column. This is

straightforward, as we can add a two-dimensional (2D) translation vector with respect to the reconstructed S/Br sub-lattice

$$\vec{a} = \left(0, \frac{a}{2}\right), \quad \vec{b} = \left(\frac{b}{2}, 0\right) \tag{5}$$

for a crystal rotation of $\theta = 0°$. For a finite rotation angle θ, we obtain the rotated coordinates

$$\vec{a}' = R(\theta) \cdot \vec{a}, \quad \vec{b}' = R(\theta) \cdot \vec{b} \tag{6}$$

with the rotation matrix defined as

$$R(\theta) = \begin{pmatrix} \cos\theta & -\sin\theta \\ \sin\theta & \cos\theta \end{pmatrix} \tag{7}$$

After rotation, the coordinates of the crystal vectors become

$$\vec{a}' = \left(-\frac{a}{2}\sin\theta, \frac{a}{2}\cos\theta\right), \quad \vec{b}' = \left(\frac{b}{2}\cos\theta, \frac{b}{2}\sin\theta\right) \tag{8}$$

4. Automated and dynamic drift compensation.

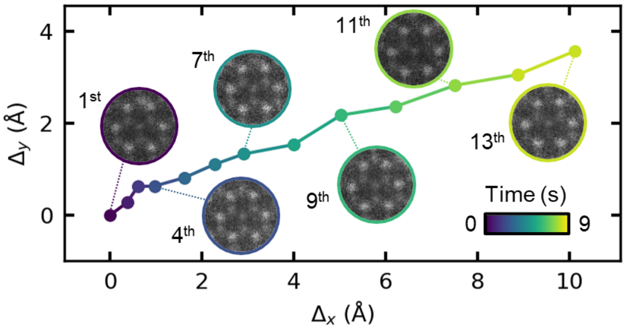

Figure S5. **Automated tracking of a single V dopant atom in 1L WS_2.** Repeated ALO on a single V dopant atom in 1L WS_2. ALO compensates a maximum drift rate of $2\,\text{Å}\,\text{s}^{-1}$.

5. Dose-Effectiveness of Atomic Lock-On.

Besides spectroscopically probing atoms, reconstructing the lattice by ALO allows sub-atomic placement with the ability to target bonds or other high symmetry sites in the crystal. We demonstrate this with 16L

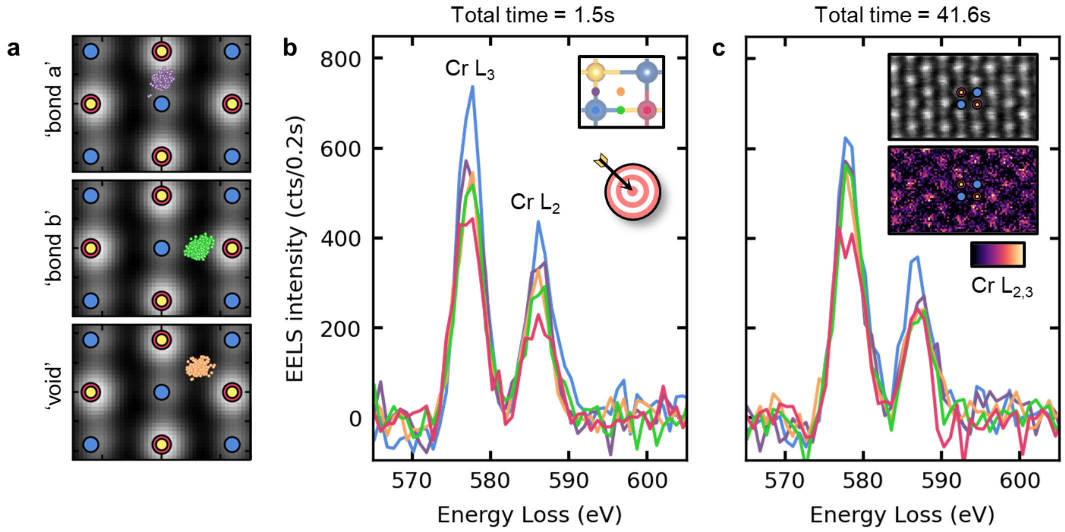

Figure S6. **Automated single-shot and targeted EELS on atoms and bonds in CrSBr a**, ALO targeting precision of the 'a bond', 'b bond' and 'void' in CrSBr with 20 ± 10pm, 19 ± 12pm, and 20 ± 10pm, respectively. **b**, Targeted single-shot EELS acquired on five target sites (see inset) of the Cr $L_{3,2}$ edge. Each spectrum is background subtracted to highlight the relative edge intensity. The integration time was 200 ms for each spectrum with a beam energy of 100 keV and a beam current of 20 pA. **c**, Regular raster scan spectral image with a total integration time of 41.6 s. 20 individual spectra from each target site are summed to reach the same total dwell time as in single-shot experiments in **b**. Inset: Simultaneously acquired HAADF-STEM, and an EELS 2D map of the Cr $L_{3,2}$ edge integrated from 570 eV − 590 eV after background subtraction. A beam energy of 100 keV and a beam current of 20 pA were used.

CrSBr by targeting the center of the bond along the a- and the b-direction and the 'void' (**Fig. S6c**). This can have applications where particular beam-driven reactions are governed by beam placement. Verifying the high targeting precision we continue performing single-shot EELS focusing on the Cr $L_{3,2}$ edge as it provides the strongest signal contrast, plus Cr atoms reside in their own atom column. **Figure S6d** shows spectra for five target sites with an integration time of 200 ms each. As expected, the Cr column shows the highest intensity and the S/Br the lowest with a contrast of almost 50%. The presence of Cr signal throughout all positions is from scattering due to the specimen thickness. The total measurement time to collect all five spectra is 1 s and only 0.5 s for performing all ALOs.

We furthermore emphasize the dose-effectiveness of targeted spectroscopic measurements by comparing with a regular pixel-by-pixel (85x49) spectral image EELS with a size of (2x1 nm^2), with total integration time of 41.6 s and a single spectrum acquisition time of 10 ms. From each of the equivalent target sites we extract and sum 20 spectra to match the total integration time for single-shot EELS and use the same background subtraction to obtain the Cr $L_{3,2}$ edge (**Fig. S6e**). We obtain the expected intensity, however,

the measurement time to collect the same information makes targeted EELS > 40 times more dose efficient compared to collecting a full spectral image, dramatically reducing electron exposure to the specimen.

6. Atomic Lock-On Precision for 1L WS_2

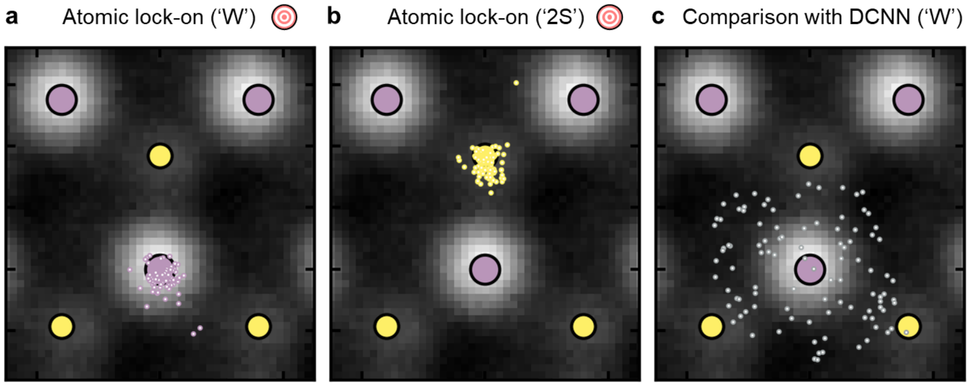

Figure S7. **ALO precision for targeting W and 2S atom columns on 1L WS_2. a, b**, ALO targeting of the W atom and 2S atom column with 28 ± 18pm ad 27 ± 17pm precision, respectively. **c**, Comparison to targeting the W atom column using a DCNN.

7. Additional Single-Atom Time Dynamics.

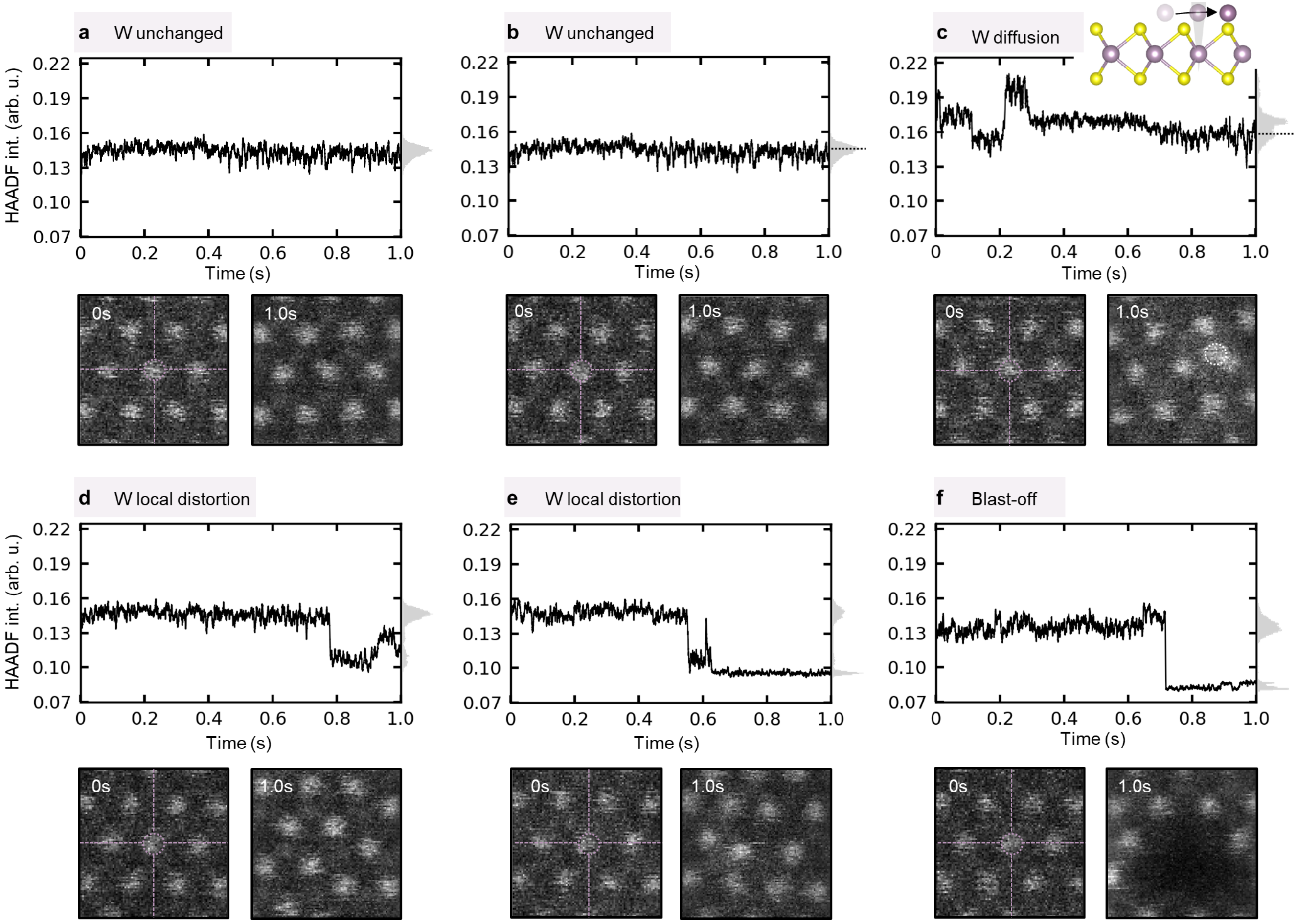

Figure S8. **Single-atom dynamics targeting W in 1L WS_2. a, b**, Unchanged crystal structure after 1s of targeting W atom column. **c**, Adatom diffusion with a HAADF intensity in excellent agreement with a W atom. In the final frame after the exposure a W atom is visible in close proximity. **d, e**, Beam induced local lattice distortions result in in-plane shifts of the W atom out of the electron beam. **f**, Beam induced material removal ('blast off') creating a nanopore. A beam energy of 60 keV and a beam current of 20 pA was used corresponding to $2.5 \cdot 10^8 e^- \text{Å}^{-2} s^{-1}$.

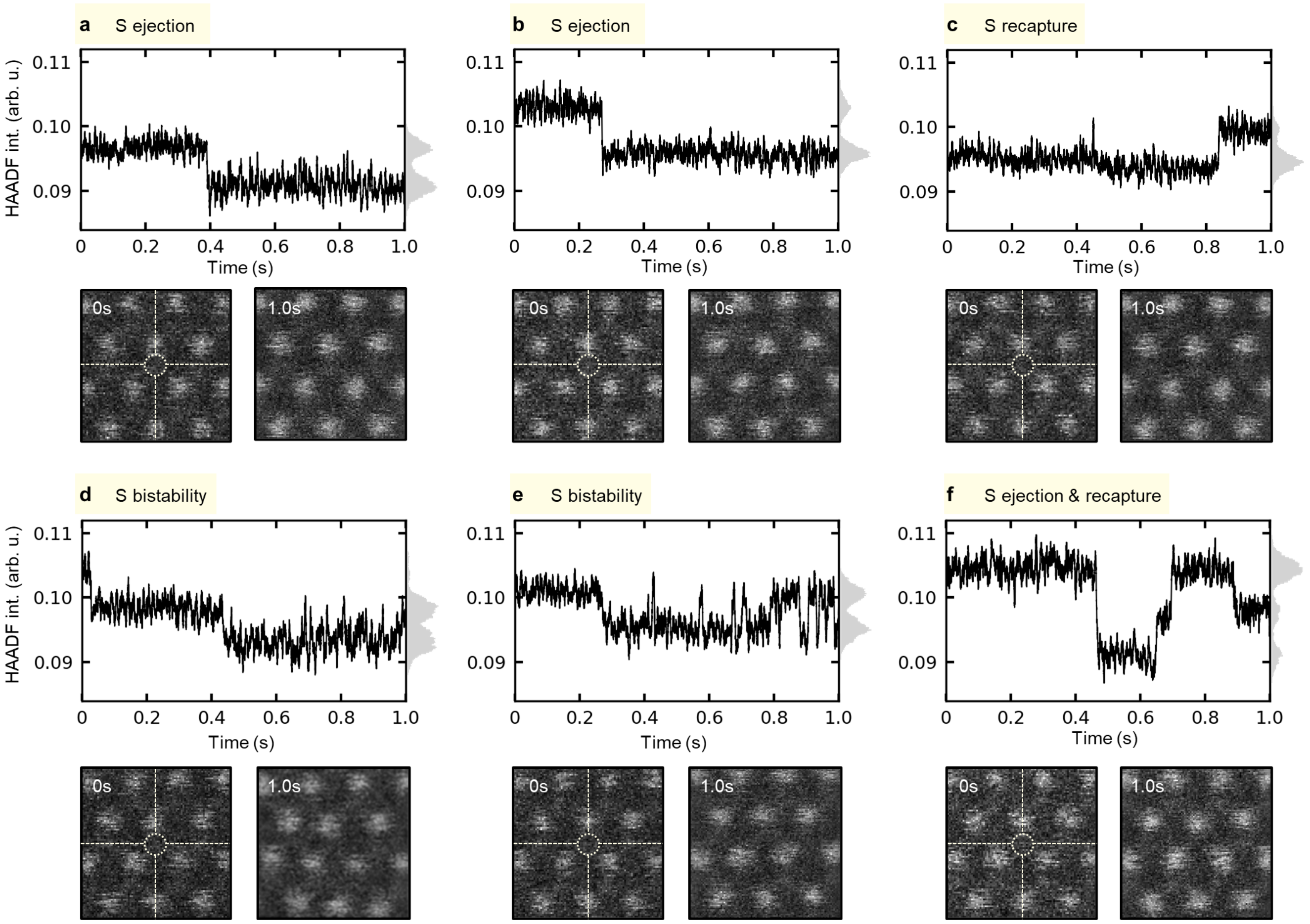

Figure S9. **Single-atom dynamics targeting 2S in 1L WS_2. a, b**, Ejection of a S atom. **c**, Recapture of a S atom. **d, e** Random telegraph noise as a signature of bistability of a S atom moving between the exposed site and a proximal position. **f**, Ejection and recapture of S atoms. A beam energy of 60 keV and a beam current of 20 pA was used corresponding to $2.5 \cdot 10^8 e^- \text{Å}^{-2} s^{-1}$.

* roccapriorkm@ornl.gov

† jpklein@mit.edu

[1] A. B. Yankovich, B. Berkels, W. Dahmen, P. Binev, S. I. Sanchez, S. A. Bradley, A. Li, I. Szlufarska, and P. M. Voyles, Nature Communications **5** (2014), URL https://doi.org/10.1038/ncomms5155.
